# Systematic Development of a Dutch School-Based Sexual Prejudice Reduction Program: an Intervention Mapping Approach

**DOI:** 10.1007/s13178-017-0301-1

**Published:** 2017-11-02

**Authors:** Fraukje E. F. Mevissen, Gerjo Kok, Anita Watzeels, Gee van Duin, Arjan E. R. Bos

**Affiliations:** 1 Department of Applied Social Psychology, Maastricht University, Postbox 616, 6200 MD Maastricht, the Netherlands; 2 Rotterdam-Rijnmond Public Health Service, Schiedamsedijk 95, 3011 EN Rotterdam, the Netherlands; 3 Department of Educational Science and Teacher Training College, University of Amsterdam, Postbox 19268, 1000 GG Amsterdam, the Netherlands; 4 Faculty of Psychology and Educational Sciences, Open University of the Netherlands, Postbox 2960, 6401 DL Heerlen, the Netherlands

**Keywords:** Sexual prejudice, Homophobic bullying, Intervention mapping, School-based intervention, Adolescents, Theory and evidence based

## Abstract

Experiences of sexual prejudice threaten the quality of life and psychological well-being of sexual minority youth. The aim of this paper is to provide a comprehensive overview of how we developed a theory- and evidence-based sexual prejudice reduction program suitable for the Dutch high school context, guided by the intervention mapping approach (IM). In line with IM, six steps were followed: an initial needs assessment in which empirical, theoretical, and new data were gathered to acquire a thorough understanding of the problem (step 1); the formulation of program objectives for both students and teachers (step 2); the selection of theory-based methods and applications (step 3); program development (step 4); the provision of an adoption and implementation plan (step 5); and the development of an evaluation plan (step 6). In conclusion, developing a sexual prejudice reduction program for schools is a challenging but feasible process. IM is an effective tool for the systematic (theory- and evidence-driven) development of such a program.

## Introduction

Although acceptance of homosexuality is slowly increasing in several Western countries (Smith, 2011), prejudiced attitudes and responses towards sexual minorities such as lesbians, gay men, and bisexual (LGB) people are still in evidence (Ahmed & Jindasurat, 2014; Herek & McLemore, 2013). For example, a recent meta-analysis (including 164 studies published between 1992 and 2009 with samples representing 18 countries and different age groups) showed that 55% of LGB individuals have at some time experienced verbal harassment while 41% have at some time experienced discrimination (Katz-Wise & Hyde, 2012). Even more worrisome are studies showing that a large number of these offenses against sexual minorities seem to occur at school (Willis, 2004)—a place which is supposed to be a safe environment for an adolescent. A recent national survey in the USA showed that 71.3% of LGB youth reported frequently hearing remarks such as “dyke” or “faggot” at school (Kosciw, Greytak, Bartkiewicz, Boesen, & Palmer, 2012). Research in other western countries such as in Europe show similar negative experiences among LGB youth in the school context (FRA, 2013; Magić & Maljevac, 2016). Experiencing sexual prejudice can contribute to physical and mental health problems such as cardiovascular diseases, depression, and anxiety disorders among sexual minorities (Baams, Beek, Hille, Zevenbergen, & Bos, 2013; Hatzenbuehler et al., 2014; Kuyper & Fokkema, 2011; Meyer, 2003). Higher victimization-related rates of depressive symptoms and suicide have also been found among sexual minority youth as compared to their heterosexual peers (Burton, Marshal, Chisolm, Sucato, & Friedman, 2013).

While the Netherlands is one of the more tolerant countries regarding acceptance of homosexuality (Collier, Horn, Bos, & Sandfort, 2015; Lottes & Alkula, 2011; Smith, 2011), it is still the case that LGB people are not completely accepted. Data from a national sample show that although only a minority of the Dutch population (5%) scores negatively on general attitudes towards LGB people, still 22% of the population does not accept same-sex marriage (Van Bergen et al., 2010). In a sample of 1600 Dutch LGB adolescents and young adults, the vast majority (75%) stated having experienced anti-gay sentiment in the 12 months preceding participation in the study (Van Bergen et al., 2010). In addition, a recent publication by Van Bergen and colleagues showed that up to 63.9% of Dutch LGB youth reported suicidal ideation, with victimization being the strongest predictor (Van Bergen, Bos, Van Lisdonk, Keuzenkamp, & Sandfort, 2013). Altogether, these data illustrate just how necessary interventions to reduce sexual prejudice among adolescents are.

There is an increasing interest in anti-prejudice interventions within the school context. Two well-known and recently evaluated approaches are the so called Gay-Straight Alliances (or GSA: extracurricular school clubs which encourage LGB and other students to meet and organize activities, often assisted by teachers, creating a safe and non-judgmental school environment), and the personal story method (whereby volunteers from the LGB community visit schools to share their coming out and personal life experiences). Both approaches have shown mixed results in terms of changing students’ and teachers’ attitudes and behaviors towards LGB youth (Eick, Rubinstein, Hertz, & Slater, 2016; Steck & Perry, 2016; Swanson & Gettinger, 2016). In addition to these approaches, the United Nations has published a practical guide for the development and implementation of anti-homophobic bullying interventions to be used by the educational sector (UNESCO, 2012). However, a systematic approach to the development of these kind of programs is still lacking and (sexual) prejudice reduction interventions often miss a clear theoretical foundation (Bartoş, Berger, & Hegarty, 2014; Bos, Pryor, Reeder, & Stutterheim, 2013; Parrott, 2008). In addition, clear step-by-step guidance on how to develop such interventions—as well as detailed descriptions of interventions that are suitable for the school context—is needed.

Research shows that the effectiveness of an intervention is promoted if its components are grounded in evidence and theory, and if it is systematically developed (Albarracín et al., 2005; De Bruin, Viechtbauer, Hospers, Schaalma, & Kok, 2009). The intervention mapping (IM) approach could provide some useful guidance for synthesizing research and integrating it with theory (Bartholomew et al., 2016). IM has proved to be an effective approach for the design of numerous behavior change interventions (See, e.g., Bos, Schaalma, & Pryor, 2008; Leerlooijer et al., 2014; Mevissen, Ruiter, Meertens, Zimbile, & Schaalma, 2011; Van Oostrom et al., 2007). The aim of our paper is to provide an example of how a prejudice reduction intervention can be developed in a systematic way, and how theory and evidence can be applied in the different stages of intervention development. In this paper, IM was used to guide the development of an intervention designed to reduce sexual prejudice in a Dutch high school setting.

IM provides program planners with a systematic framework for effective and theory-based decision-making at each of the six steps specified in the developmental process. Program development starts with a needs assessment (step 1) which focuses on a thorough problem analysis, resulting in a “logic model of the problem.” This includes a description of the health-related problem; quality-of-life impact; the at-risk population; and social-cognitive, behavioral, and environmental factors (and their determinants) thought to be related to the problem. Next, planners select target groups and formulate objectives for change at the behavioral level and at the social-cognitive level, based on importance and changeability (step 2). In step 3, planners select theory-based methods and strategies which are then integrated into the final program (step 4). Program adoption and implementation is described in step 5 and the program evaluation plan is produced in the last step (step 6).

At each step, the IM approach encourages program planners to pose planning problems as questions and to get the answers by brainstorm, searching for empirical evidence, and using theory or conducting new research if evidence is lacking. Intervention Mapping clearly distinguishes between theories that can be used to *explain* behavior and theories that can be used to *understand how to change* behavior (Kok et al., 2016). Theories explaining behavior provide guidance in terms of *what* should be changed. Theories explaining how to change behavior provide guidance in identifying techniques that can be used to bring about changes in the determinants that explain the behavior. There are also theories that can be used to explain both the behavior and how best to change it. A good example of this type of theory is Allport’s contact hypothesis (Allport, 1954) that explains why some people are less prone to stigmatize than other people. At the same time, it explains under which circumstances contact can help to reduce stigmatizing attitudes and behaviors.

As well as using theory, IM encourages the researcher to analyze the problem and to formulate change not only at an individual level but to also consider the different levels in the environment. It is important to note that IM is an iterative process, and that the information collected in one step will guide the decisions made in subsequent steps. IM encourages program planners to consider the adoption and implementation of the program from the start of the developmental process by creating a linkage group; i.e., a planning group including future program adopters, implementers, and other important stakeholders in the community.

It is not only important to systematically develop a program, but also to provide a detailed description of the program and its developmental process. A precise and systematic description promotes program replication, larger-scale dissemination, and ideas for improvement (Peters, De Bruin, & Crutzen, 2015). If the theories underpinning the program as well as the program components and activities are clearly set out, it can guide and enhance future work in the field of sexual prejudice reduction. To redress this issue, we do not present the results of the effects of our intervention in this paper, but instead explicitly focus on a detailed description of how we systematically developed a school-based sexual prejudice reduction program.

## Methods and Results

### The Dutch Educational System

The Dutch educational system (EP-Nuffic, 2015) is divided not only in terms of public or private schools and primary and secondary education, but also in terms of educational level (different schools and types of study programs can be chosen on the basis of a child’s individual results and capacities). After completing primary education (ages 5–12), students will enroll in one of three types of secondary education: “preparatory secondary vocational education” (VMBO—for students 12 to 16 years old, which prepares students for secondary vocational education (MBO for students 16–19 years old)), or the higher general educational levels of HAVO (“higher general education”—for students 12 to 17 years old, trajectory of 5 years) or VWO (“pre-university training”—for students 12 to 18 years old, trajectory of 6 years). The Dutch government formulates learning objectives for all subjects within each study program. Formerly, schools were not obliged to include sex education in their teaching activities, although many did to some extent, mainly focusing on the biological aspects as part of the Biology curriculum. As of December 2012, however, the objectives include sections on sexuality and sexual diversity (SLO, 2015). This means that all schools in the Netherlands are required to incorporate these topics into their curriculum. But teachers have freedom in terms of how they organize their lessons and which study materials they select.

### Study Setting and Background

In 2005, the Dutch NGO’s STI Aids Netherlands (www.soaaids.nl) and Rutgers (Knowledge Center on Sexual and Reproductive Health and Rights: http://www.rutgers.international/) published their report on the sexual health of youth in the Netherlands called “sex below 25” (seks onder je 25ste; De Graaf, Meijer, Poelman, & Vanwesenbeeck, 2005). This report, including an action plan, was presented during a meeting that several important stakeholders had been invited to, including other sexual health oriented NGOs, health care workers, scientific researchers, and members of the government. The action plan recommended the revision of the well-known and widely used school-based Dutch sexual health program Long Live Love (LLL) for youth aged 12–14 (Schaalma et al., 1996), and additionally called for the development of sexual health programs for older students targeted to their specific needs. In addition, it was considered important that sexual diversity should be addressed in all sexual health programs. Based on these recommendations, the Dutch Ministry of Health, Welfare and Sport provided financial support for the development of school-based sex education geared towards the different types of secondary education (VMBO, MBO, and HAVO/VWO). The sexual prejudice reduction program described in this paper was developed for older students (age 15–17) enrolled in higher level education (HAVO/VWO) and was part of a larger-scale school-based program about sexual health called Long Live Love + (LLL+; see Mevissen et al. (in press). All six steps of intervention mapping (IM) were followed. In this section, we will describe, step-by-step, exactly how we developed our program and what the results were.

### Step 1 Needs Assessment

The first step was to acquire a thorough understanding of the problem of sexual prejudice among adolescents, its consequences, its determinants, and the actors involved. The needs assessment was guided by the PATH-model (Buunk & Van Vugt, 2013) and the PRECEDE-PROCEED model (Green & Kreuter, 2005). We carried out a thorough problem analysis by searching the literature, brainstorming with stigma research experts, and performing additional research. Key actions were the identification of the following: health issues and quality-of-life problems associated with sexual prejudice, the at-risk population (i.e., those being directly and negatively affected; in this case, those being stigmatized—the sexual minority youth), relevant behavioral and environmental factors and agents, and key determinants related to these behaviors.

#### The At-Risk Population

Sexual minorities include a broad range of people with different variations in sexual orientation and gender identity. Although there are similarities in the (often low) quality of life and (high) prejudice experiences between subgroups, each subgroup also has to deal with unique quality of life issues and prejudice experiences (Fredriksen-Goldsen, Kim, Barkan, Balsam, & Mincer, 2010; Kertzner, Meyer, Frost, & Stirratt, 2009). In addition, determinants explaining prejudiced responses towards one group can be different from those explaining responses towards the other subgroups (Van Alphen, Dijker, Bos, Van Den Borne, & Curfs, 2011). The development of a program that focuses on the broader range of all sexual and gender identities, taking into account all these differences, would be too expensive and time consuming, not only for the developers but also for those implementing the program (the teachers). Therefore, in the program described in this paper, we mainly focused on the subgroup of lesbian, gay, and bisexual (LGB) individuals. Although we are fully aware of the limitations of this decision, recent studies have shown that prejudice reduction towards one subgroup can transfer to a reduction in prejudice towards other similar groups (Harwood, Paolini, Joyce, Rubin, & Arroyo, 2011; Pettigrew, 2009). Moreover, it has been shown that a general stigma-reduction technique can be similarly effective in reducing prejudice towards different subgroups (Vezzali, Stathi, Giovannini, Capozza, & Trifiletti, 2015).

As described in the Introduction, multiple studies show that LGB youth still experience different forms of discrimination and stigmatization, especially in the school context, and that this negatively influences their well-being (Katz-Wise & Hyde, 2012; Van Bergen et al., 2013). Experiencing negative reactions can, for example, result in depression or anxiety disorders (Hatzenbuehler et al., 2014; Herek & Garnets, 2007; Mustanski, Newcomb, & Garofalo, 2011), and result in self-stigma (Bos et al., 2013). Sexual prejudice is thus predominantly a problem for those directly involved, namely (young) LGB individuals. However, when the prejudice experiences of LGB individuals leads to serious health problems, it may also become a problem for others in their environment (partners, family, friends, and colleagues) and for society in general (e.g., in terms of the financial costs of psychological treatment and unemployment).

Research shows that many young LGB individuals struggle with coming out and with accepting their sexual identity (Rosario, Schrimshaw, & Hunter, 2004). It has also been shown that LGB individuals can suffer from internalized sexual prejudice (Hatzenbuehler, Nolen-Hoeksema, & Dovidio, 2009; Meyer, 2003). On the other hand, available support (e.g., from family and friends) and having adequate coping styles have both been related to health and well-being among LGB people (Meyer, 2003).

#### Who Are Involved?

In the school context, the well-being of LGB individuals is often negatively influenced by seeing or experiencing homophobic bullying (name-calling, negative comments, exclusion; Collier, Bos, & Sandfort, 2013; FRA, 2013). The literature on school-based bullying behavior (Olweus, 1996; Olweus & Limber, 2010; Saarento, Garandeau, & Salmivalli, 2015; Stassen Berger, 2007) identifies four different actors in school-based bullying: (1) the bullies or perpetrators. These are individuals who repeatedly attack another individual who does not fight back. Most bullies feel powerful and secure. In an average school or class, they are in the minority, but they are powerful, have influence, and are difficult to change. (2) The victim—the individual who suffers from repeated attacks of bullying. It has been suggested that there are two types of victims: passive victims (who are weak and defenseless) and bully-victims (who are aggressive and who are not only victims of bullies but also bullies themselves). In an average class, victims are also often in the minority. (3) The observers or bystanders. Often, bystanders do not dare to stand up to bullies or speak out against bullying because they are afraid of becoming the victim. They usually form the majority in the school or classroom. An important intervention strategy is thus to mobilize the bystanders into becoming defenders. (4) The adults or teachers. These are the people who can make a difference by actively intervening when they observe bullying taking place and by providing a “good example.”

Research has shown that if a school has no anti-bullying or anti-discrimination policy, or is not actively implementing such a policy, a LGB-safe environment cannot be created (Bos et al., 2013; Olweus & Limber, 2010). In addition, a lack of implementation of (theory- and evidence-based) anti-prejudice interventions, or a lack of the financial means to develop such interventions may contribute to the problem of sexual prejudice. Finally, if LGB individuals lack social support from peers, parents, or teachers, it is more difficult for them to stand up to the prejudice they encounter. Together, these findings suggest that teachers have a dual role in reducing sexual prejudice namely both as targets and as implementers of an intervention. Interventions should target teachers directly as their behavior needs to change in order to create LGB-safe school environments. In relation to the development of our program, this meant that, in step 2, we formulated objectives for the teachers. As teachers also play a role in adopting and implementing the prejudice-reduction intervention designed to target the students, we developed an adoption and implementation plan for teachers in step 5.

#### What Causes Sexual Prejudice?

The scientific literature provides an extensive list of individual determinants that have been shown to be directly related to sexual prejudice: age, gender, religiousness, educational level, ethnicity, contact, gender role beliefs, masculinity, authoritarianism, attribution, motivation to control prejudice, social norms, and affect (emotions) (see, e.g., Collier, Bos, & Sandfort, 2012; Haider-Markel & Joslyn, 2008; Herek, 2000; Horvath & Ryan, 2003; Olson, Cadge, & Harrison, 2006; Parrott & Gallagher, 2008; Smith, Axelton, & Saucier, 2009; Tsang & Rowatt, 2007). Support for these determinants is also reflected in theories and frameworks on (sexual) stigma and prejudice (see, e.g., Bos et al., 2013; Herek, 2007). However, the main body of scientific literature on (determinants of) sexual prejudice is based on studies conducted in the USA with a focus on homosexuality, and studies among adults or university students. Moreover, while many different determinants influencing sexual prejudice have been described, the relative importance of these factors remains unclear. Therefore, as part of the needs assessment, we performed a cross sectional study among 636 Dutch adolescents (*M*age = 16.3 (SD = 2.2), 51.1% girls). For reasons of homogeneity (see our earlier description on diversity between sexual minority subgroups), we decided to focus only on negative intentions towards homosexual individuals as the outcome measure and not specifically used lesbians and bisexuals in the formulation of the questions. Relative importance analyses were then performed in order to select the most important determinants involved in sexual prejudice (Johnson & Lebreton, 2004).

The results showed that homo-negative attitudes such as the opinion that homosexuality is not normal turned out to be the strongest predictor for having negative behavioral intentions towards homosexual individuals. In addition, lack of contact with homosexual individuals, attributing homosexuality to something learned instead of something that is innate, and more negative emotions towards public expressions of homosexuality (kissing, walking hand-in-hand) were all strongly related to prejudiced responses. For a more comprehensive report on individual determinants and their relative importance in influencing sexual prejudice, see Mevissen et al. Correlates of sexual prejudice among Dutch adolescents: A relative importance approach (under review). Based on these analyses, the following most important and changeable determinants influencing sexual prejudice were selected to be targeted (in 15+ high school students enrolled in higher level secondary education) by the sexual diversity program: attitude, contact, attribution, and affect (emotions).

Based on the findings of step 1, we decided to focus on the following three program goals: (1) reduction of sexual prejudice, including homophobic bullying, among high school students; (2) teachers and students creating a safe school environment; (3) increased well-being among LGB students.

### Step 2 Matrices of Change Objectives

Together with a multidisciplinary linkage group, objectives were formulated in order to attain the program goals defined at the end of step 1. At this stage, the linkage group consisted of 22 participants: two stigma research experts, four applied psychology researchers (including the principal investigator (PI)), one social worker, one public health worker, and 14 teachers. Together with the researchers, the social worker, and the public health worker, the PI defined desired changes for the target population at the behavioral level (i.e., performance objectives, PO).

As no examples of systematically developed sexual prejudice-reduction interventions were available in the literature to guide the development of the objectives, the project team decided to look again into the literature addressing school-based bullying behavior and prevention (Olweus, 1996; Olweus & Limber, 2010; Saarento et al., 2015; Stassen Berger, 2007). The work by Olweus on bullying behavior in schools showed that only a comprehensive approach targeting different levels simultaneously (students, teachers, schools, parents) can be effective in changing bullying (Olweus & Limber, 2010). This literature on bullying behavior and prevention inspired the project group to create different POs and separate matrices for different actors involved in school-based sexual prejudice: the perpetrators, the bystanders, the victims (i.e., LGB youth), and the adults (i.e., teachers). Table 1 presents the POs formulated in relation to each actor for our sexual prejudice reduction intervention. As evident from this list, the POs for the bullies are restricted to the prevention of bullying (e.g., *respect sexual diversity* and *leave sexual minorities alone*), while those for the bystanders are more directed towards active involvement (e.g., *report sexual prejudice*). We expected the perpetrators to be the most strongly prejudiced against homosexuality and to be the most difficult to change. Trying to make the perpetrators befriend LGB individuals is thus not a realistic goal and the number of POs for them was therefore limited. The concept of “respect” and being respected—or being able to be yourself—is a very important issue for adolescents, and may be something more easily taken on board by perpetrators than a request to “accept sexual diversity.”

**Table 1:** Performance objectives for each actor (perpetrators, bystanders, victims, teachers)

Actor	Performance objectives
Perpetrators	P.1 Respect sexual diversity
	P.2 Leave LGB peers (or those you think may be LGB) alone. [No attention is better than negative attention]
Bystanders	B.1 Report sexual prejudice or bullying to a teacher/confidential advisor
	B.2 Stand up for LGB peers
	B.3 Cope with feelings of insecurity and discomfort related to homosexuality and bisexuality
	B.4 Support LGB peers, e.g., during coming out
Victims	V.1 Young LGB students mobilize (social) support if needed.
	V.2 Young LGB students cope with feelings of discomfort, insecurity, doubt, etc. related to their sexual identity
	V.3 Young LGB students cope with negative responses in the environment regarding their sexual identity
Teachers	T.1 Intervene in case of (LGB-related) discrimination and bullying in or around school
	T.2 Support young LGB individuals

Furthermore, we developed POs for LGB individuals with the aim of increasing well-being by helping them to cope with sexual prejudice and self-stigma. We formulated three POs in relation to LGB students. The first is to mobilize (social) support if needed. Research has shown that LGB individuals who are supported by others suffer less from the consequences of sexual prejudice (Kwon, 2013; Masini & Barrett, 2008; Ryan, Russell, Huebner, Diaz, & Sanchez, 2010). Other studies have found that feeling supported by family or friends or feeling part of a community of peers results in better quality of life (Kertzner et al., 2009; Meyer, 2003; Mustanski et al., 2011). The second PO for LGB students is to cope with feelings of discomfort related to their sexual identity (Rosario et al., 2004). The third PO for LGB students is to cope with the sexual prejudice they encounter in their environment. As we did not expect to be able to completely eradicate sexual prejudice, it seemed relevant to also provide tools for the LGB students to help them cope with prejudiced responses.

Finally, the POs for the teachers focused on intervening (e.g., take action when bullying is noticed) and providing support for young LGB students. The studies conducted by Olweus (1996; Olweus & Limber, 2010) and Honig (2006) have shown that, in order to effectively deal with bullying, it is important to change the entire school climate and involve stakeholders from both inside and outside the school. Unfortunately, however, at the time that this sexual prejudice program was developed, changing the entire school climate was beyond the scope of our project (due to time and money restrictions). Note that the final program integrated all objectives for the students (i.e., perpetrators, bystanders, and victims), as it was not the intention of the project team to develop separate interventions for each subgroup of students and teachers. Our description of step 4 will explain in more detail how all objectives for the different actors were combined into one intervention.

After formulating the POs, determinants related to each PO had to be selected. The selection of determinants was based on theories of stigma, theories of health behavior, and the relative importance analyses conducted as part of our needs assessment. The selection was not only based on the importance of a specific determinant for a specific PO, but also on its changeability. For example, the determinant “social norm” was expected to have a big influence on the PO “Leave LGB peers (or those you think may be LGB) alone,” especially within a school context. However, the literature shows that changing social norms is relatively difficult (Mollen, Ruiter, & Kok, 2010), and we therefore excluded social norms. On the other hand, attitudes are also strongly related to sexual prejudice (i.e., important) but have been shown to be relatively easier to change, so they were included as a target determinant for change.

After determinants for each PO had been selected, the objectives for change at the social-cognitive level could be formulated. Changes objectives (COs) were formulated by matching each determinant with its specific PO, after which the COs content were guided by the needs assessment. For example, in relation to the PO for perpetrators “Leave LGB peers (or those you think may be LGB) alone,” we selected “attitude” as one of the determinants. For this specific attitude–PO combination, we formulated the CO “perpetrators acknowledge the importance of not giving negative attention to LGB peers,” as the needs assessment showed that perpetrators of bullying behavior are difficult to change—so this CO seemed to be the most achievable option. Likewise, in relation to the PO for bystanders “Report sexual prejudice or bullying to a teacher or a confidential advisor,” we selected “skills” as one of the determinants, and the related CO was formulated as “Describe how you recognize (sexual) prejudice and bullying,” as the needs assessment showed that bullying behavior can be difficult to recognize as it often happens in a hidden way (Marston, 2015; Stassen Berger, 2007). Table 2 presents more examples of POs, determinants, and CO combinations in a selection of the matrices (full matrices will be provided via https://www.researchgate.net/project/Long-Live-Love-A-Dutch-School-Based-Online-Sexual-Health-Program-for-Adolescents-aged-15). The goals and objectives were discussed and fine-tuned in collaboration with the members of the linkage group. During two focus group interviews, feedback provided by the teachers was used to finalize the matrix.

**Table 2:** Examples of the matrices (summary) for the four different actors targeted in the sexual prejudice reduction program (perpetrators [P], bystanders [B], victims [V], and teachers [T]) including performance objectives [PO] for each group, determinants, and change objectives [CO]

Actor and PO	Determinants and CO				
Perpetrators	Knowledge	Attitude	Affect	Self-efficacy	Skills
P2 Leave LGB peers (or those you think may be LGB) alone	Give examples of what bullying is and what discrimination is Describe the (psychological) short- and long-term consequences of bullying and excluding	Acknowledge the importance of not giving negative attention to LGB peers Acknowledge that you can just politely say “no” to a same-gender proposal, just like you can to an opposite-gender proposal	Acknowledge that contact with LGB individuals is not a threat to your own sexual orientation or image Acknowledge that being bullied or excluded is not nice		
Bystanders	Knowledge	Attitude	Affect/stigma-by-Association	Self-efficacy and self esteem	Skills
B1 Report sexual prejudice or bullying to teacher/confidential advisor	Describe different manifestations of discrimination or bullying Mention that equal treatment, regardless of ethnicity, religion, gender, or sexual orientation is constitutionalized in the Netherlands; discrimination is prohibited by law	Acknowledge the importance of reporting discrimination and bullying	Acknowledge that standing up for a LGB peer does not mean you are lesbian, gay or bisexual Recognize the emotions experienced by somebody being bullied	Feel confident in reporting discrimination/bullying	Describe how you recognize (sexual) prejudice and bullying Describe which steps to take when wanting to report discrimination/bullying
Victims	Knowledge	Attitude	Affect	Self-efficacy and self esteem	Skills
V1 Young LGB individuals mobilize (social) support if needed.	Give examples of problems for which one may ask for support Give three examples of people or organizations who/that can provide reliable support or information	Acknowledge the importance of looking for support Mention (dis)advantages of (not) looking for support	Recognize emotions that may be part of looking for support (negative, but in the end positive)	Are confident in looking for support	Describe how you can ask for support
Teachers	Knowledge	Attitude	Affect	Self-efficacy	Skills
T1 Intervene when discrimination or bullying occurs in or around school	Describe the (psychological) short- and long-term consequences of bullying and exclusion for the victim	Describe the pros and cons of intervening when bullying/discrimination occurs	Express empathy for LGB individuals	Express confidence in intervening	Describe how to cope with the disadvantages of intervening

The complete matrices include 37 COs for the perpetrators, 47 COs for the bystanders, 28 COs for the victims (young LGB individuals), and 31 COs for the teachers, and are available via https://www.researchgate.net/project/Long-Live-Love-A-Dutch-School-Based-Online-Sexual-Health-Program-for-Adolescents-aged-15

Unfortunately, and again due to time and money restrictions, at the end of step 2, the program developers concluded that it was not possible to develop an intervention or training program specifically for the teachers in order to target the POs and COs that were formulated to change their behavior. According to the teachers, this was not too much of a problem because they already felt sufficiently able to intervene in cases of bullying and were confident that they could support LGB students.

### Step 3 Selection of Theory-Based Methods and Applications

Having defined objectives for change, theoretical methods were selected from the literature. Methods were then translated into practical applications appropriate for the school-based context of the program. The selection of methods and applications was an iterative process: methods were selected from the literature and, following a brainstorming session, were translated into a potentially feasible practical application. However, the opposite process also took place: existing stigma-reduction programs were reviewed for their applicability in our sexual diversity program. If applicable, the program was related to theoretical methods. If necessary, adjustments were made such that the application was fully in line with the evidence-based theoretical method. We asked for feedback from the teachers on these methods and applications in a focus group interview.

Our sexual diversity program was developed as part of a larger school-based program on sexual health called LLL+ (see Mevissen et al. (in press)). LLL+ was originally planned to be delivered online. However, teachers from the linkage group made clear that, particularly in relation to certain topics like homosexuality, classroom interaction was important. They therefore preferred classroom-based assignments to online tasks. In addition, teachers stressed that classes can differ greatly in terms of their group dynamics. Some classes can be very quiet and contain many shy students; other classes can easily become very restless and therefore need more structure. Group work and activities or discussion could thus work very well in one class but cause chaos in another. It is also worth noting that the teacher must provide firm guidance during interactive assignments—something not all teachers feel comfortable with, especially in relation to sexuality-related topics. A final important recommendation from the teachers was to carefully consider time constraints in the Dutch high school setting; one class lasts 45 min on paper, but this leaves 35 min of real-time teaching, as students need time to change classrooms, get seated, and get organized.

The project team therefore selected methods and applications that would fit the context in which the sexual diversity program would be used. This meant that the final program should be flexible in use, easy to adjust to different classroom circumstances, and mainly comprised of off-line assignments. In addition, our target group included pre-university students; the program should therefore be mentally challenging enough to match the target groups’ mental capacities. Also, the program would need a comprehensive and detailed teacher manual to guide the teacher in performing the lessons. Table 3 provides an overview of the program including methods and applications and how they relate to the determinants and COs.

**Table 3:** Overview of the sexual diversity program including scope and sequencing related to objectives (Summary), Methods, Parameters, Strategies and Materials

Lesson	Assignment	Determinants and change objectives (summary)	Methods	Parameters	Applications	Materials
1. Visibility, prejudices and facts	1. Circle exercise	*Knowledge* Mention that you cannot always see if somebody is lesbian, gay or bisexual (LGB)	Active Learning	Needs time, information and skills.	A circle is drawn on the blackboard (see Figure 4). Students are encouraged by the teacher to mention LGB men and women they know within the different levels of their environment (family, school, media) and place their names in the circle. The teacher makes clear that it is not the goal to ‘expose the gay person’ in this exercise or to provide private information on personal family situations if that is not wanted. The teacher guides the discussion by pointing out that it is easier to come up with visible, famous and openly LGB men and women but that there are many LGB men and women that are ‘unknown’. Examples of LGB celebrities that are less visible are provided. To conclude, the teacher guides the discussion towards the wide diversity seen in relation to LGB individuals (just as in relation to heterosexual Individuals).	Teacher manual with instructions and background information to guide the discussion. See Figure 3.
			Discussion	Listening to the learner to ensure that the correct schemas are activated.		
			Elaboration	Individuals with high motivation and cognitive ability; messages that are personally relevant, surprising, repeated, self-pacing, not distracting, easily understandable.		
		*Stereotyping* Mention that homosexuality is expressed in different ways (behavior, lifestyle), just like heterosexual individuals express themselves in different ways	Stereotype inconsistent information	Only effective when there are many different examples and examples that are not too discrepant from the original stereotype.		
	2. Sexual Diversity: terminology	*Knowledge* Mention the difference between transgender, transvestism and homosexuality and that being transgender is also nature (not nurture).	Active learning Elaboration Discussion	See above. See above. See above.	The different terms that students may associate with sexual diversity will be explained. The teacher will present a term and ask the students to explain what it is. If unknown or if a wrong definition is given, the teacher will make a correction or ask another student to respond.	Power point presentation including terms and their meanings.
		Mention that many adolescents struggle with their sexual identityMention that many adolescents struggle with their sexual identity			Additional information can be provided by the teacher, for example on the prevalence of homosexuality and the fact that many people can sometimes feel attracted towards same-gender individuals without being homosexual.	Teacher manual with instructions and background information to guide discussion.
		*Empathy* Express empathy for the difficulties associated with not being accepted or with being stigmatized (and not feeling comfortable with your gender for transgender adolescents)	Empathy training	Requires being able and willing to identify with the stigmatized person. Imagine how the other would feel (this leads to empathy). Do not imagine how you would feel because this also leads to distress.	To round off, a video can be shown about a transgender girl (see Figure 2). In the video, the girl explains her struggle with being in a female-body while feeling male. But it is also about the support she received from her family.	Video with personal story of transgender girl.
		*Knowledge* Give three examples of people or organizations that can provide reliable support or information	Advanced organizers	Schematic representations of the content or guides depicting what is to be addressed.	A brochure will be handed out to the students by the teacher at the end of the lesson. The brochure includes a summary of the discussed terms. In addition, it includes a list of websites where students can find more information on sexual diversity or LGB support groups.	Brochure with summary of the terms discussed and websites with additional information.
	3. Nature or nurture?	*Knowledge, awareness* Mention that homosexuality is nature and not nurture (attribution) Mention that homosexual men would not prefer to be women, and lesbian women would not prefer to be men: there is no standard gender-role division in all homosexual couples.	Active learning	See above.	Four statements will be discussed by the students: 1) Homosexuality has always existed and can be found in all cultures 2) In the Netherlands, homosexual individuals are allowed to get married and have or adopt children 3) Homosexuality is nature 4) A homosexual man actually prefers to be woman; a lesbian woman actually prefers to be man. If the situation allows, the discussion can first take place in small groups before summing up with the whole class. It is preferable to mix up the students (in terms of sexual preference, olerance for sexual diversity and cultural background) so that different opinions can be heard. The teacher will guide the discussion such that in the end, the conclusions will be in favor of the first three statements and in disagreement with the last statement.	PowerPoint presentation with statements. Teacher manual with instructions and background information, including new arguments to guide the discussion.
			Discussion	See above.		
			Elaboration	See above.		
			Arguments	For central processing of arguments to occur, they need to be new to the message receiver.		
2. Homo-sexuality nearby	4. Coming out	*Knowledge* Mention organizations where discrimination can be reported, both anonymously and confidentially.	Discussion Active Learning Elaboration	See above. See above. See above.	The teacher explains what ‘coming out’ means. After that, four questions need to be discussed and answered by the students: 1) What could be disadvantages of coming out for a young homosexual individual? 2) What could be seen as advantages of coming out? 3) What could be the consequence of leading a ‘secret homosexual life’ 4) Living as a homosexual individual in a heterosexual society: how would that be? If the situation allows, the questions can first be answered in small groups before summing up with the whole class. It is preferable to mix up the students (in terms of sexual preference, tolerance for sexual diversity and cultural background), so that different opinions can be heard.	PowerPoint presentation with questions.
		*Attitude* Acknowledge that ‘being (able to be) yourself’ also applies to LGB individuals	Arguments	See above.		
		*Empathy* Express empathy for the difficulties associated with not being accepted or with being stigmatized	Empathy training	See above.		
			Shifting perspectives	Initiation from the perspective of the learner, needs imaginary competence.		
		*Affect* Mention the emotions related to not being accepted.	Dramatic relief	Preferably should occur within a counseling context so that emotions can be aroused and subsequently dealt with.		
		*Skills, self-efficacy* LGTB youth mention how they can cope with negative responses in the environment	Planning coping response	Identification of high-risk situations and practice of coping response.	The teacher will guide the discussion to include how to deal with certain difficulties associated with coming out. In the end, the teacher will guide the discussion into the direction of showing the importance of everyone being able to be him/herself. That it is brave for young people to come out of the closet, and that this should be respected.	Teacher manual with instructions and background information to guide discussion.
		LGTB youth feel supported by important others	Guided practice	Sub-skill demonstration, instruction, and enactment with individual feedback; requires supervision by an experienced person; some environmental changes cannot be rehearsed.		
		LGTB mention three organizations/people where convenient information and support can be found	Mobilize social support	Availability of social network and potential support givers.	A brochure will be handed out to the students by the teacher at the end of the lesson. See description above.	Brochure with LGTB network and support groups.
		LGTB youth are confident in looking for support	Modeling	Attention, remembrance, self-efficacy and skills, reinforcement of model; identification with model, coping model instead of mastery model.	A video can be shown in which a girl talks about her positive (at school) and negative (in the church) experiences associated with coming out. The video can be shown before the questions are posed as a warming-up exercise or at the end as a way of wrapping up.	Video with personal coming out story of lesbian girl including positive and negative experiences.
	5. Coming out at school	*Knowledge* Mention that the coming-out process differs per person and can have both advantages and disadvantages.	Active learning Discussion, Elaboration, Arguments	See above. See above. See above. See above.	Four questions/statements need to be discussed and answered by the students (see below). If the situation allows, the questions/statements can first be answered/discussed in small groups before summing up with the whole class. It is preferable to mix up the students (in terms of sexual preference, tolerance for sexual diversity and cultural background), so that different opinions can be heard.	PowerPoint presentation with questions and statements.
		*Awareness* Mention the (psychological) short and long-term consequences of bullying and sexual prejudice	Consciousness raising	Can use feedback and confrontation; however, raising awareness must be quickly followed by an increase in problem-solving ability and (collective) self-efficacy.	1) At our school, an homosexual boy or girl can come out without any problem.	Teacher manual with instructions and background information to guide discussion.
		*Affect, Empathy* Mention emotions when being bullied or sexual prejudiced	Environmental reevaluation	May include awareness about serving as a role model for others.		
			Empathy training	See above.		
		*Social influence* Acknowledge own influence on preventing sexual prejudice and bullying	Information about others’ approval	Positive expectations are available in the environment.	2) What can you do when you see a peer being bullied because of his/her sexual orientation?	Teacher manual with instructions and background information to guide discussion.
		*Self-efficacy, skills* Feel confident in reporting sexual prejudice and bullying	Modeling	See above.		
		*Awareness* Describe how you recognize sexual prejudice and bullying	Self-reevaluation	Can use feedback and confrontation; however, raising awareness must be quickly followed by an increase in problem-solving ability and self-efficacy.		
		*Awareness, Attitude* Acknowledge that important others do not assume you are lesbian, gay or bisexual when standing up for LGB individuals	Consciousness raising, Self-reevaluation, Environmental reevaluation, Shifting perspectives	See above. See above. See above. See above.	3) Those who have homosexual friends are homosexuals themselves.	Teacher manual with instructions and background information to guide discussion.
		*Self-efficacy, skills* Describe different ways to support a friend in coming out	Mobilizing social support, Planning coping response	See above. See above.	4) How can you support a friend in coming out?	Teacher manual with instructions and background information to guide discussion.
		*Self-efficacy, skills*	Guided practice	See above.	The teacher will guide the discussion such that solutions for how to deal with difficult situations will also be discussed.	Teacher manual with instructions and background information to guide discussion.
		*Attitude, Affect* Describe emotions that can play a role in supporting a friend	Shifting perspectives	See above.	The teacher will also ask more questions in order to elaborate on topics – for example: ‘How would you do that?’; ‘Would it be different at school vs. in the classroom?’; ‘How would you feel?’.	

More elaborate information on the theories and references behind the methods included in this table can be found in Bartholomew, L. K., Markham, C. M., Ruiter, R. A. C., Fernández, M. E., Kok, G., & Parcel, G. S. (2016). Planning Health Promotion Programs. An Intervention Mapping Approach (4th ed.). San Francisco: Jossey-Bass

To target, for example, the CO “mention that homosexuality is nature and not nurture,” which relates to the determinant “knowledge,” we selected the methods “active learning,” “discussion,” and “elaboration.” All three methods are expected to change knowledge in a long-lasting way (theories of information processing, elaboration likelihood, see Petty, Barden, & Wheeler, 2009). However, in order for these methods to be effective, several parameters need to be taken into account (Kok et al., 2016). Active learning requires time for the learner to elaborate on the information provided and respond to it, instead of only consuming information without thinking about it at a deeper level. In addition, the method “discussion” requires that somebody (e.g., the teacher) listens to the learners to ensure that the correct schemas (i.e., existing repertoires of information in peoples mind that in this case should be positively directed towards the CO) are activated. The applications for the methods that the project team proposed would take those parameters into account. In this kind of set-up, rather than just giving students a text to read explaining that homosexuality is nature and not nurture (passive learning), a statement is introduced and the students are challenged to respond to it (active learning). Students are given time to think about the statement and form their own ideas and discuss these with each other (parameter “time”). The teacher should guide the discussion such that in the end, the correct information is activated (parameter “listen to the learner”). See Table 3 for more examples.

### Step 4 Program Development

The program development was guided by the selection of methods and applications in step 3. The final selection of program components and the order in which they appear was guided by topic and by time constrains: Due to the sensitivity of the topic, the project team found it important to sequence the assignments in the program starting with more basic, factual information, moving on to argumentation, and finishing with more person-directed and affective discussions. In this way, the teacher has more time to create a safe atmosphere, find out how the students will respond, and build up confidence among students in relation to discussing the topic. At the same time, different activities had to fit into the 45-min high school teaching schedule and not exceed a maximum of two teaching hours in total. We asked for feedback from the teachers on the final program components. When doubts about the practical application of a program component arose (e.g., related to time constraints or how understandable it might be for the students), that specific component was pilot-tested among students in the classroom by one or several of the teachers and—if necessary—the component was adjusted. Table 3 provides an overview of the program in terms of scope, sequencing, and materials.

The final program consists of five assignments divided over two teaching hours or lessons. The first lesson includes three assignments which introduces the topic of sexual diversity, provides some background information and facts, and focuses on breaking down stereotypical ideas about homosexuality by targeting the determinants knowledge, awareness, empathy, and subjective norms. The second lesson includes two assignments addressing coming out, first introduced from the perspective of the LGB-person (“how would it be?”) and, in the next assignment, from the perspective of the bystanders (“what can YOU do to make your school/class a bully-free environment?”). This lesson focuses more on attitudes, empathy, affect, self-efficacy, and skills.

**Fig. 1:**
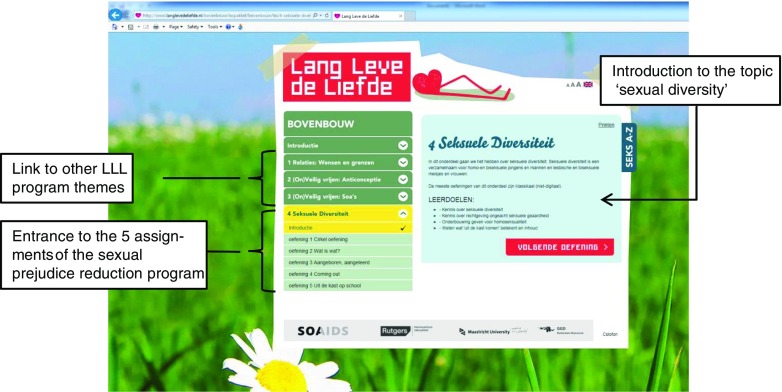
Screenshot of the Long Live Love (LLL) website (student section), including the sexual prejudice reduction program theme

**Fig. 2:**
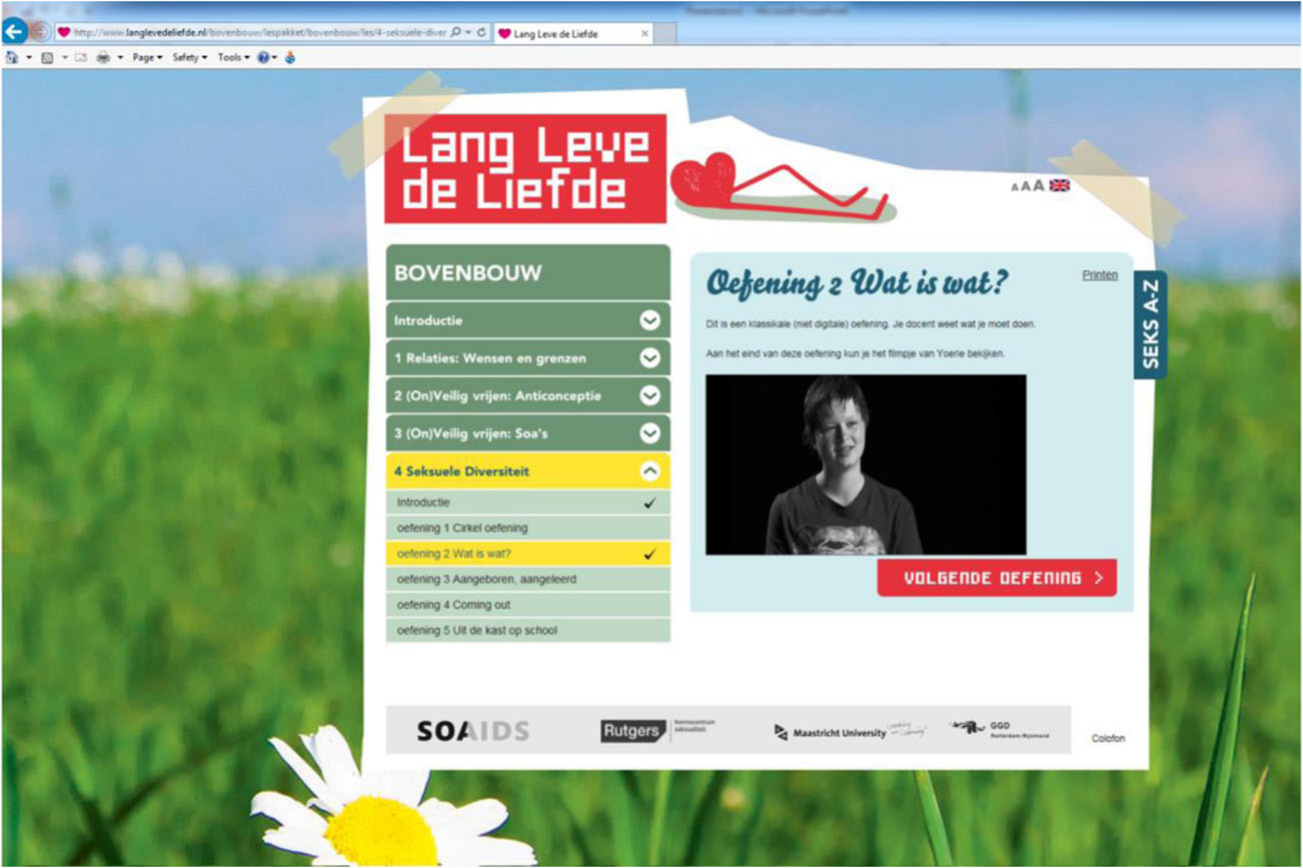
Screenshot of the webpage for assignment 2 of the sexual prejudice reduction program

**Fig. 3:**
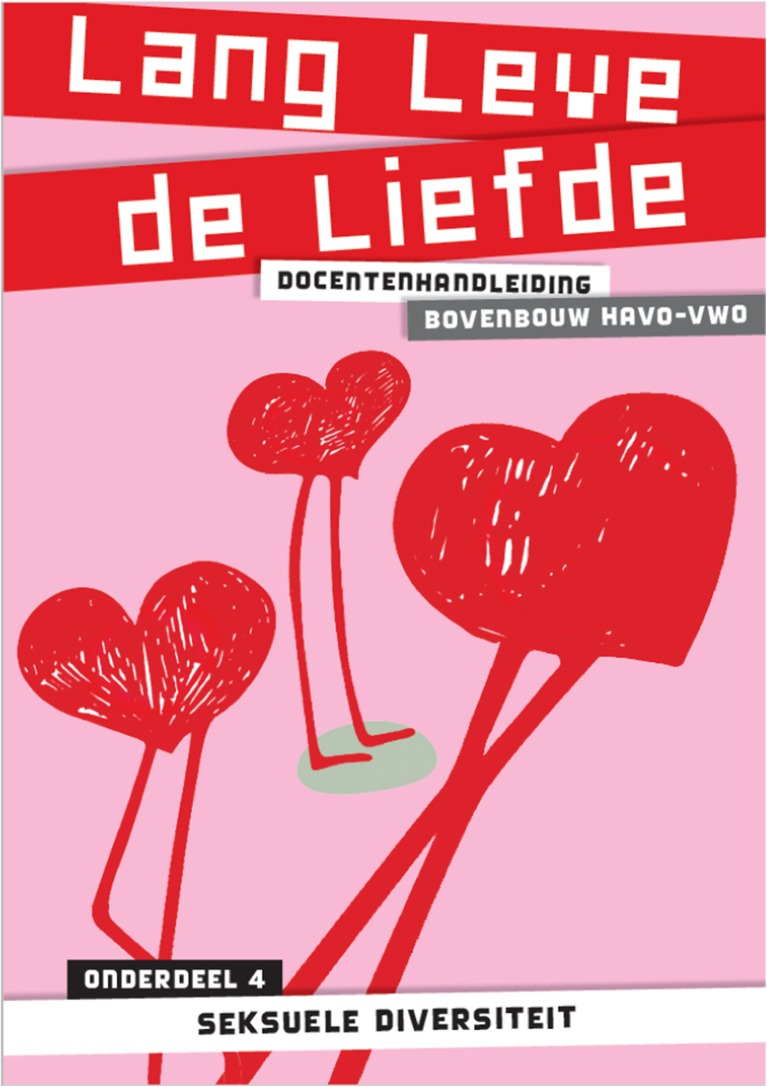
Front page of the teacher manual

The main program components developed by the project team were the teacher manual, three PowerPoint presentations and a brochure. The PowerPoint presentation can be used by the teachers to support or guide the discussions. The brochure was designed as a general hand-out for all students and includes information regarding sexual diversity. However, the underlying purpose of the brochure is to provide (in a confidential manner) LGB students—or those students who are still uncertain of their sexual identity—with information on LGB community and support websites. The two videos included in the program were adopted from COC (a Dutch LGBT organization; www.coc.nl). All materials are provided for free via the website that was developed as part of the larger school-based sexual health program (see www.langlevedeliefde.nl; ‘Bovenbouw’ section, see also Fig. 1). The website includes a section for teachers and a section for students (Figs. 1 and 2). The section for teachers contains background information on (teaching) the topic of sexual health and sexual diversity. It also refers to an e-coaching website specifically developed to support teachers in teaching sexual health (see step 5 and Schutte, Van Den Borne, Kok, Meijer, & Mevissen, 2016). In addition, the section for teachers provides the teacher manual (see also Fig. 3), which can be downloaded free of charge, as well as the PowerPoint presentations. The student section explains the different assignments to the students and includes the videos (see, e.g., Fig. 2). These program components were all discussed and fine-tuned together with all linkage group members.

### Step 5 Adoption and Implementation

**Fig. 4:**
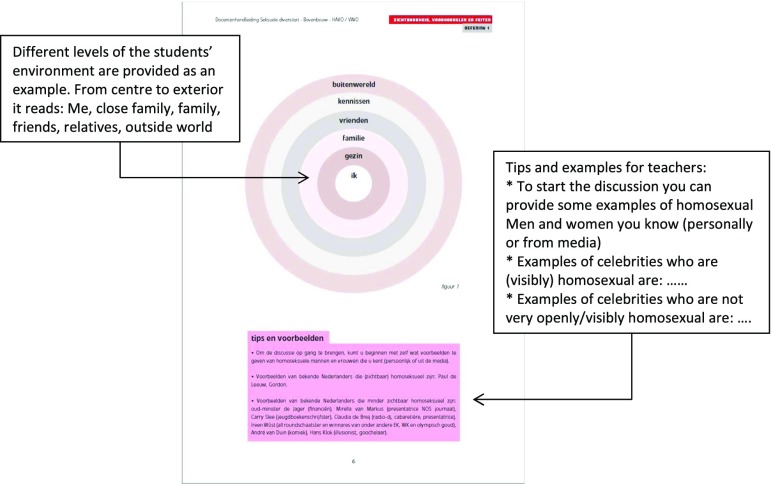
Instructions in the teacher manual related to assignment 1 (circle exercise)

As well as consulting the literature on adoption and implementation, we used feedback from the teachers to guide all steps of program development and the production of the teacher manual (Fig. 3). In addition, we expected that ownership would be created by closely involving teachers right from the very start of the program development. In the Netherlands, topics related to sexual health are usually taught by Biology teachers. However, Biology is not a mandatory subject for our target group of 15+ HAVO/VWO students. To guarantee that all students would receive the sexual diversity program, it was necessary that the program could also be adopted and implemented by non-Biology teachers. We therefore attempted to recruit teachers with different teaching backgrounds into our linkage group. In the end, the linkage group included 14 teachers from different high schools; 12 Biology teachers and two social studies teachers. At this stage, the literature on adoption and implementation of school-based sexual health programs was reviewed (e.g., Paulussen, Kok, & Schaalma, 1994; Wiefferink et al., 2005). Based on the literature, and the teachers’ feedback and suggestions regarding conditions for use of the sexual diversity program, adoption, and implementation POs were formulated and determinants for these POs were selected. The main PO was that teachers would implement the complete sexual diversity program (i.e., implement ALL assignments) and that teachers would implement the program with fidelity (i.e., implement the program according to the guidelines provided in the teacher manual). To achieve this PO, the project team selected three determinants to target as part of program development in order to improve completeness and fidelity of implementation of the sexual diversity program: *organizational constraints*, *outcome beliefs*, and *teacher benefits*. The aim of the project group was to influence these determinants, partly by adapting the program ideas (i.e., (number of) program objectives, program applications, etc.) such that it would fit the teachers’ context and overcome any barriers they mentioned, and partly by including guidance via the teacher manual (Fig. 4).

Regarding *organizational constraints*, the teachers had made it clear that the program had to be flexible. Each school and each class setting is different and therefore requires a different approach. For example, not all schools have sufficient computer and internet facilities. Thus, the program was developed in such a way that all assignments could also be implemented off-line (thus enhancing completeness). Another potential constraint is that group-work or individual assignments do not always work well in each class. To deal with this barrier, the teacher manual provides multiple suggestions in relation to each assignment. In this way, the teacher can select the strategy that best fits the specific class-context, without having to skip an assignment (influencing completeness) or having to adjust an assignment (influencing fidelity).

An important *outcome belief* for teachers is the importance of the student learning outcomes and whether students like the lessons (Paulussen et al., 1994; Wiefferink et al., 2005). The student learning outcomes (i.e., performance and change objectives) were formulated by the project team. When necessary, these learning outcomes were adjusted based on the teachers’ feedback (while still following the IM guidelines). In this way, the final objectives were in line with the teachers’ outcome beliefs. Moreover, the assignments were developed and adjusted based on the teachers’ feedback regarding their experience in terms of what students may like. An emphasis was placed on the student learning outcomes in the teacher manual. Furthermore, a program evaluation plan was described and planned (see step 6) in order to gather additional information that could further improve adoption and implementation.

From the beginning of the project, it became clear that the teachers’ main concern was related to time. The teachers in the linkage group stressed repeatedly that they have a very busy schedule, with many competing targets and too little teaching hours to include everything that they would like to. This time constraint, which was not mentioned in the literature, is probably explained by the fact that our target group is high school students who are entering their graduation years (final exams). Teachers thus experience a lot of pressure to fulfill all official learning objectives as stated by the government. It was clear for the project team that these time constraints (*teachers benefit*) had to be taken into account. We used several strategies in order to accomplish this.

First of all, a trade-off had to be made between striving for completeness (including all COs) on the one hand, and fitting everything into a limited number of teaching hours (2 h) on the other. Having a program that needed too many teaching hours would be a serious threat for its adoption. This meant that a selection had to be made in terms of which COs to include in the final program. The decision-making process with regard to which COs to exclude was partly guided by the literature (theory and evidence) on the importance and changeability of the determinants and COs in question. Additionally, our aim was to include a logical and comprehensive selection of topics in the final program. Together, these decision-making processes resulted in, for example, leaving out COs related to social norms, as these are more difficult to change (Mollen et al., 2010), as well as focusing the exercises on the topic of homosexuality and only limited on bisexuality and transgender issues. This was a difficult decision; however, we were reassured by the findings of Peters and colleagues showing that developing skills and positive attitudes in one behavioral domain seems to be transferable to other domains as well (Peters, Ten Dam, Kocken, Buijs, Dusseldorp, and Paulussen, 2015).

Next, the teacher manual included a brief but very clear stepwise description of the exercises, including a proposed timetable. Our intention was to limit any preparation time that the teachers needed. It also includes suggestions for homework assignments; making students prepare part of the assignments at home further reduces the time needed during teaching hours. The teacher manual also recommends which assignments should receive priority (assignments 4 and 5)—particularly important if the teacher is short of time. Hopefully, this will guarantee that at least the most important objectives will be targeted. While we are aware that the latter suggestion is not in line with having the program implemented completely, we are also aware of the need to be pragmatic. Throughout the entire developmental process, teachers repeatedly emphasized their lack of time to us. We therefore concluded that it was better to provide guidance in terms of which exercises to prioritize in situations where their time is very limited. Finally, and probably most importantly, another strategy we used to enhance adoption and implementation was to ensure that the objectives of the program matched the learning objectives as formulated by the government. At the time of developing our sexual diversity program, the learning objectives for Biology were being updated and reformulated. One of the teachers in our linkage group was a member of the committee that contributed to the reformulation of the learning objectives concerning the topic of sexuality. The objectives formulated for LLL+ by the project team were adopted by this committee and subsequently by the government. In addition, the committee included the Long Live Love program, including the sexual diversity program described here, in the list of recommended school programs that could be implemented to fulfill the learning objectives as formulated by the government.

The teachers in the linkage group said that they did not need support in teaching sexual diversity (in terms of self-efficacy, skills, etc.), but that it could be an issue for other teachers. We did not have funds available to develop further supporting materials for teachers (e.g., the provision of teacher training) as part of the sexual diversity program. The project team therefore decided to refer teachers to an e-coaching website that was developed as part of another project (Schutte et al., 2016). The aim of this e-coaching website is to enhance the adoption and implementation of the LLL program for students aged 12–14. It includes strategies targeting self-efficacy and skills for teaching sexual diversity.

### Step 6 Program Evaluation

For the last step in the IM protocol, a plan was made for program evaluation. This evaluation plan included a description of the effect evaluation measures as well as criteria for a process evaluation. In planning for the evaluation of the effectiveness of the programs in reducing (determinants of) sexual prejudice, outcomes for the students (effect evaluation) as well as outcomes for the implementers, the teachers (process evaluation) were identified. For the students, it was decided that the performance objectives as formulated in step 2—as well as the determinants and change objectives as described in the matrices—would be adequate evaluation measures and preferable to the use of standardized, more general measures of sexual prejudice. The project group recommended testing the effectiveness of the programs among a wider sample of Dutch high schools using a randomized controlled trial. For the process evaluation, it was decided that interviews with both teachers and students should be conducted, focusing on their assessment of the program and factors influencing completeness and fidelity of program implementation. The actual evaluation of the sexual prejudice reduction program took place as part of a pilot implementation of the entire LLL+ program. The reports on these evaluations are currently in progress.

## Discussion

In this paper, we describe the systematic development of a Dutch sexual prejudice reduction program for high school students. This sexual prejudice reduction program was developed as part of a larger sex education program, focusing on different aspects of sexual health: the Long Live Love+ (LLL+) program for senior high school students aged 15+ (in Dutch Lang Leve de Liefde Bovenbouw. See www.langlevedeliefde.nl). The aim of this sexual prejudice reduction program is to reduce negative behavior towards lesbian, gay, and bisexual (LGB) individuals, mobilize social support, and strengthen the coping skills of LGB teenagers. Research on (reduction of) sexual prejudice so far lacks a tradition in systematic intervention development (Bartoş et al., 2014). This paper provides an example of how the systematic development of a theory- and evidence-based intervention program is possible. The intervention mapping framework (IM) was used to guide program development. IM turned out to be a useful tool to structure the planning of the intervention, integrating insights from different experts, theories, empirical studies, and target group members.

Applying each of the different steps of IM provided useful insights which then guided subsequent steps. As part of the needs assessment (step 1), additional data were gathered on determinants of sexual prejudice among Dutch high school students. These data provided information on the relative importance of each of the determinants (Mevissen et al. Correlates of sexual prejudice among Dutch adolescents: A relative importance approach under review)).—findings that guided the decisions made regarding the intervention objectives in the subsequent step (step 2). Another important contribution in terms of shaping the intervention objectives in step 2 were the insights gained from reviewing the literature on bullying behavior (Olweus, 1996; Olweus & Limber, 2010; Saarento et al., 2015; Stassen Berger, 2007). Some aspects of prejudiced behavior are closely related to bullying behavior, for example name calling or neglect. Theories explaining bullying behavior among adolescents distinguish between four different actors; the perpetrators, the bystanders, the victims, and the adults (Olweus, 1996; Stassen Berger, 2007). This distinction helped us to create different matrices including different objectives for the different actors involved in sexual prejudice-related bullying situations. Designing the final program (step 4) partly overlapped with selecting methods and strategies (step 3). Both steps were guided by feedback provided by the teachers involved in the linkage group as well as by examples of strategies that have been used by others in attempts to reduce sexual prejudice. Factors that could influence the adoption and implementation of the program were considered by discussing the outcomes of each step with the teachers in the linkage group. A teacher manual was provided to motivate and guide teachers in terms of implementing the program completely and with fidelity (step 5). Finally, an evaluation plan—focusing on the effects of the intervention as well as its implementation—was written (step 6).

In our view, the multidisciplinary linkage group was vital to the development of the program. The input from different experts not only guided theory- and evidence-based decisions, but also resulted in many creative solutions being put forward. The input from the teachers was extremely influential in terms of shaping the final intervention into a program that would be feasible for them to implement, i.e., a program flexible in use, that can be adjusted to suit different school contexts and class-dynamics, and, importantly, one that is not too time consuming. Although actual levels of adoption and implementation of the sexual prejudice reduction program have not yet been evaluated, it is likely that both the existence of official governmental policies requiring inclusion of the topic of sexual diversity in the school curriculum and the fact that teachers were involved in program development will help in enhancing program implementation (Swanson & Gettinger, 2016; UNESCO, 2012).

Although the systematic development of the sexual prejudice program was in many ways successful, some shortcomings should be mentioned. First, it turned out to be very difficult to recruit non-Biology teachers to join our linkage group. This may have influenced the final flavor of the program. Future research should focus on how teaching sexual health in general, and sexual diversity specifically, can also be adopted by teachers from other disciplines. If teaching sexual health and sexual diversity is limited to teachers of a specific subject, this may limit program reach. Secondly, due to time and financial constraints, not all change objectives for the students as formulated in step 2 could be targeted. For the same reasons, the performance objectives formulated for the teachers in step 2 (e.g., to intervene in instances of discrimination and bullying in or around school; see Table 1) could not be targeted at all. Moreover, and again due to time and financial limitations, it was not possible to perform an elaborative study exploring teachers’ attitudes, knowledge, skills, etc. towards implementing anti-bullying and sexual diversity policies and programs. Previous research has shown that such research can greatly enhance our understanding of the needs of teachers in creating LGB supportive school environments and the potential barriers to accomplishing this (Honig, 2006; O’Donoghue & Guerin, 2017; Pizmony-levy, 2011; Swanson & Gettinger, 2016; Taylor et al., 2016). Although we closely involved teachers in the development of our program and provided implementation guidance by means of an elaborate teacher manual, the lack of either a needs assessment study among teachers or the provision of teacher training may limit the effects of the program.

In this paper, we show how a theory- and evidence-based sexual prejudice reduction intervention for high schools was systematically developed using IM. The work described in this paper could provide several academic, social, and public policy implications. First of all, with our example, we hope to guide future directions in sexual prejudice research and contribute to more theory- and evidence-based prejudice reduction programs. Also, the use of our program (or future programs guided by our approach) will contribute to increased health and well-being among LGB youth by providing them support and by reducing sexual prejudice among their peers. Additionally, our work may not only inspire researchers but also teachers and other people around the globe working with youth on how to develop their own prejudice reduction programs in a systematic way. Finally, this paper may guide policy makers in developing standards and guidelines for the adoption and implementation of theory- and evidence-based sexual prejudice reduction programs and interventions.
